# The elegance of prickly sensations

**DOI:** 10.7554/eLife.84161

**Published:** 2022-11-21

**Authors:** Bibi Nusreen Imambocus, Peter Soba

**Affiliations:** 1 LIMES Institute, Department of Molecular Brain Physiology and Behavior, University of Bonn Bonn Germany; 2 Institute of Physiology and Pathophysiology, Friedrich-Alexander-Universität Erlangen-Nürnberg Erlangen Germany

**Keywords:** nociception, mechanosensation, dendritic morphology, Ppk1/Ppk26, Piezo, Ca-α1D, *D. melanogaster*

## Abstract

Neurons sensing harmful mechanical forces in the larvae of fruit flies have a striking architecture of dendrites that are optimized to detect pointy objects.

**Related research article** Liu Z, Wu MH, Wang QX, Lin SZ, Feng XQ, Li B, Liang X. 2022. *Drosophila* mechanical nociceptors preferentially sense localized poking. *eLife* **11**:e76574. doi: 10.7554/eLife.76574.

How do we distinguish between being poked by something benign like a cotton swab and a sharp nail that could harm us? This is achieved through neurons lying beneath our skin called nociceptors which are tuned to detect painful, or noxious, stimuli (Basbaum et al., 2009). A lot of what is known about nociceptors comes from studying cells called class IV dendritic arborization neurons (or cd4a neurons for short) in larvae of the fruit fly *Drosophila melanogaster* (Grueber et al., 2002).

Protruding from the cell body of c4da neurons is a mesh of branches known as dendrites, which can sense noxious environmental cues (Tracey, 2017). This includes the ovipositor of parasitic wasps, a needle-like structure used to lay eggs in live hosts. When the larvae feel the mechanical pressure of the ovipositor poking their skin, they react by rolling away to escape being punctured by the wasp (Figure 1A). However, it is poorly understood how these sensory neurons with their astounding architecture can capture such potentially life-threatening mechanical forces.

**Figure 1.**
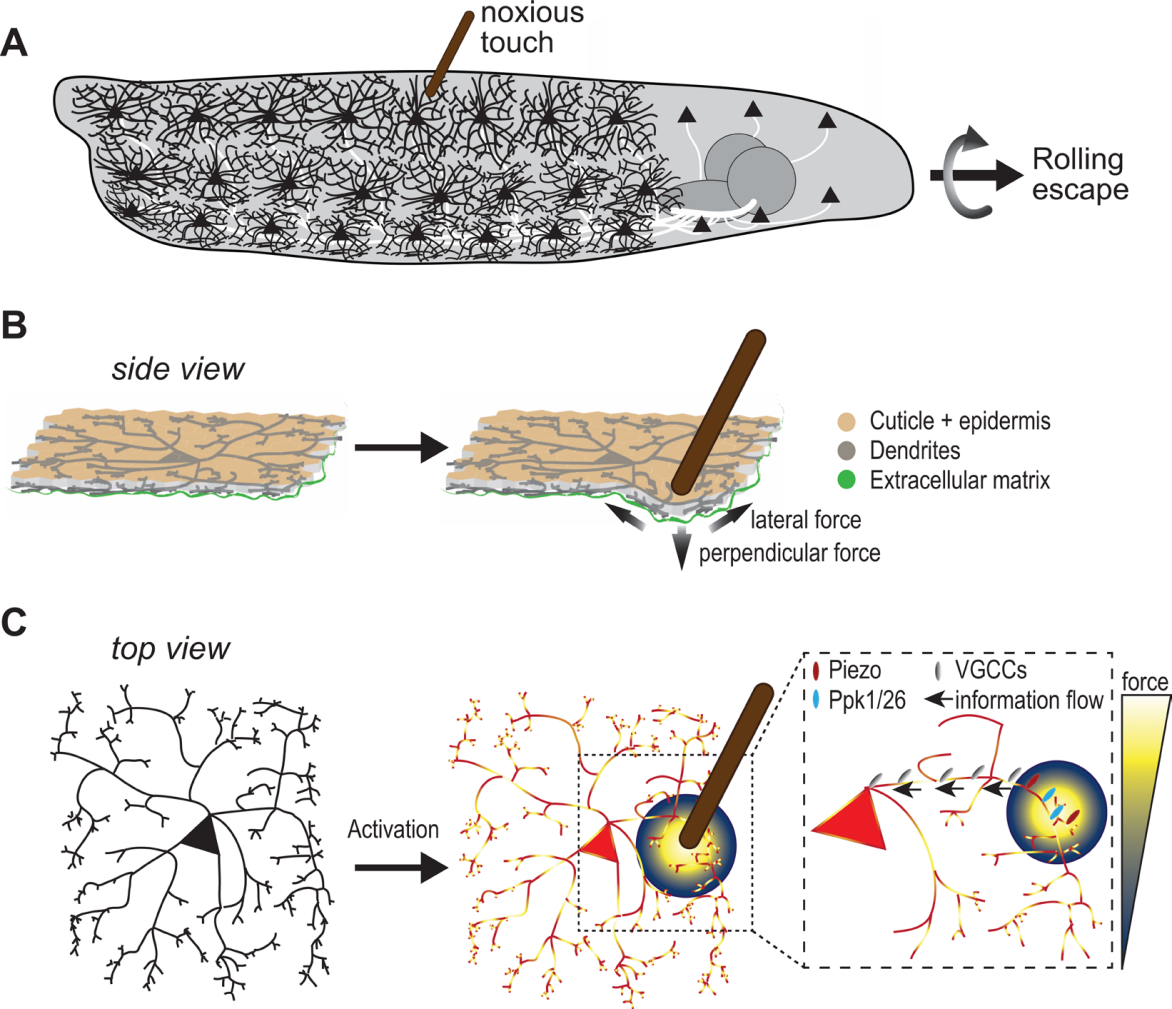
Sensing noxious touch in *Drosophila* larvae. (**A**) Schematic of a *Drosophila* larva with nociceptive cells called c4da neurons covering its entire body. The cd4a neurons can sense noxious touch (here provided by a small diameter probe) resulting in the larva undergoing a rolling escape response. (**B**) Schematic side view of c4da neurons in the larval body sandwiched between the epidermal cell layer (beige) and the extracellular matrix (green). The noxious touch of the probe deforms the body wall including c4da dendrites and generates perpendicular and lateral forces (indicated by arrows) (**C**) The force applied by the probe (yellow-blue color code) activates c4da neurons by triggering two mechanosensory channels – Ppk1/Ppk26 (cyan) and Piezo (red) – located on its dendrites. Voltage-gated calcium channels (grey) then allow information to reach the soma so they can elicit a response in the c4da neuron soma.

As shown by the famous drawings of the neuroscientist Santiago Ramón y Cajal, cells in the nervous system come in a range of shapes and sizes. One might therefore wonder whether the elegant structure of c4da neurons allows them to perform their sophisticated mechanosensing role. Now, in eLife, Xin Liang and colleagues from Tsinghua University – including Zhen Liu and Meng-Hua Wu as joint first authors – report that c4da dendrites are pressure sensors which are shaped and tuned to maximize detection of local mechanical forces (Liu et al., 2022).

The team designed and built an experimental setup to monitor the activity of c4da neurons in semi-intact preparations preserving the ‘skin’ of the larvae (containing the epithelial cell layer, sensory neurons and muscle cells) as well as their nervous system. The c4da neurons were then imaged to see how they responded to different sized probes that were applied with varying force to dendrites that were either close (proximal) to the cell body of the neuron (the soma) or were far away (distal) from the soma, of the neuron. This revealed that c4da dendrites were more sensitive to small probes (30µm), which could even be sensed by dendrites outside the local area of where the force was applied.

To find out if the dendritic architecture of c4da neurons is required for sensing localized forces, the team studied larvae lacking the gene for cut, a transcription factor that gives the neurons their complex structure. The c4da neurons of the mutant larvae were less responsive to small probes applied to their distal dendrites, and the larvae displayed reduced rolling escape behavior.

Using mathematical simulations, Liu et al. showed that their probe exerted both a perpendicular pressure at its its tip, and lateral forces up to 40µm away (Figure 1B). If neurons can sense both these lateral and perpendicular forces, this means more of their dendrites will be activated by the probe, leading to enhanced neuronal sensitivity.

To test this possibility, Liu et al. studied two mechanosensitive channels present on the membrane of c4da dendrites called Piezo and Ppk1/Ppk26 (Kim et al., 2012b; Gorczyca et al., 2014; Guo et al., 2014; Mauthner et al., 2014). Different forces with smaller or larger probes were applied to proximal and distal dendritic regions of larvae lacking the genes for Piezo and/or Ppk1/Ppk26. This revealed that both channels contribute to sensing lateral forces. In addition, Ppk1/Ppk26 is required for the overall mechanosensitivity of c4da neurons, and Piezo particularly enhances sensitivity to probes with small diameters.

Mechanosensitive channels are crucial for sensing the local stimuli applied to dendritic branches, but how is this information reaching the cell body of the neuron? Liu et al. found that voltage-gated calcium channels – which when open permit an influx of calcium – play a critical role in this process. In their experiments, low doses of a drug that inhibits these voltage-gated calcium channels strongly affected calcium responses in the neurons when the mechanical stimulus targeted dendrites further away from the soma, suggesting decreased information flow. Further experiments revealed a specific voltage-gated calcium channel called Ca-α1D is required for the mechanosensory signal to reach the soma, which also correlated with reduced escape behavior in fruit fly larvae that lacked Ca-α1D (Figure 1C).

Overall, Liu et al. show that the mechanosensing capability of c4da neurons is elegantly implemented through its dendritic structure as well as mechanosensory and voltage-gated calcium channels which maximize responsiveness to pointy objects. However, these findings bring up other questions. For one, nociceptors do not work in isolation; a mechanical force applied to a larval body wall or to mammalian skin acts on the entire tissue. In larvae, this includes an epidermal cell layer and the extracellular matrix sandwiching the mesh of c4da dendrites (Figure 1B; Han et al., 2012; Kim et al., 2012a).

Epidermal cells have a concerted influence on the mechanosensation and stability of c4da neurons (Jiang et al., 2019). Indeed, Piezo1 is present in cells of the mouse epidermis and they actively participate in sensing of mechanical stimuli (Mikesell et al., 2022). It is therefore possible that *Drosophila* epidermal cells could also aid c4da neurons. Moreover, many nociceptors (including c4da neurons) are polymodal, meaning they can detect different noxious stimuli, and they can become hypersensitive upon injury and inflammation to protect the affected body regions from further harm (Basbaum et al., 2009; Tracey, 2017). How integration of all these diverse roles is optimized by nociceptors and their surrounding tissue is certainly an exciting area for future studies.

## References

[bib1] Basbaum AI, Bautista DM, Scherrer G, Julius D (2009). Cellular and molecular mechanisms of pain. Cell.

[bib2] Gorczyca DA, Younger S, Meltzer S, Kim SE, Cheng L, Song W, Lee HY, Jan LY, Jan YN (2014). Identification of Ppk26, a DEG/ENaC channel functioning with Ppk1 in a mutually dependent manner to guide locomotion behavior in *Drosophila*. Cell Reports.

[bib3] Grueber WB, Jan LY, Jan YN (2002). Tiling of the *Drosophila* epidermis by multidendritic sensory neurons. Development.

[bib4] Guo Y, Wang Y, Wang Q, Wang Z (2014). The role of PPK26 in *Drosophila* larval mechanical nociception. Cell Reports.

[bib5] Han C, Wang D, Soba P, Zhu S, Lin X, Jan LY, Jan Y-N (2012). Integrins regulate repulsion-mediated dendritic patterning of *Drosophila* sensory neurons by restricting dendrites in a 2D space. Neuron.

[bib6] Jiang N, Rasmussen JP, Clanton JA, Rosenberg MF, Luedke KP, Cronan MR, Parker ED, Kim H-J, Vaughan JC, Sagasti A, Parrish JZ (2019). A conserved morphogenetic mechanism for epidermal ensheathment of nociceptive sensory neurites. eLife.

[bib7] Kim SE, Coste B, Chadha A, Cook B, Patapoutian A (2012a). The role of *Drosophila* Piezo in mechanical nociception. Nature.

[bib8] Kim ME, Shrestha BR, Blazeski R, Mason CA, Grueber WB (2012b). Integrins establish dendrite-substrate relationships that promote dendritic self-avoidance and patterning in *Drosophila* sensory neurons. Neuron.

[bib9] Liu Z, Wu MH, Wang QX, Lin SZ, Feng XQ, Li B, Liang X (2022). *Drosophila* mechanical nociceptors preferentially sense localized poking. eLife.

[bib10] Mauthner SE, Hwang RY, Lewis AH, Xiao Q, Tsubouchi A, Wang Y, Honjo K, Skene JHP, Grandl J, Tracey WD (2014). Balboa binds to pickpocket in vivo and is required for mechanical nociception in *Drosophila* larvae. Current Biology.

[bib11] Mikesell AR, Isaeva O, Moehring F, Sadler KE, Menzel AD, Stucky CL (2022). Keratinocyte PIEZO1 modulates cutaneous mechanosensation. eLife.

[bib12] Tracey WD (2017). Nociception. Current Biology.

